# Tetanus1Read before the annual meeting of the Pennsylvania State Veterinary Medical Association, March, 1898.

**Published:** 1898-04

**Authors:** Walker S. Phillips

**Affiliations:** Reading, Pa.


### TETANUS.[1]

By Walker S. Phillips, V.S.,
READING, PA.

This terrible disease is of nervous origin. It generally follows some operation or severe injury, and also frequently occurs from the pricking of the sole by a nail. As this affliction produces a peculiar irritability of the nervous system, it is of great importance to have the patient removed to a remote or isolated place, and kept as quiet as possible. In my treatment of these cases I have very seldom administered purgatives, but have taken advantage of bran mashes the first four to six days, to keep the bowels moving. During my thirty-eight years' practice I can recall sixteen cases, traumatic and idiopathic, which fully recovered, and none of which I placed in slings.

*Treatment.* Hypodermically, morphia, 3 grains, once daily. ℞.—Ext. belladonna, 8 drachms; laudanum, 4 ounces; chloroform, 4 ounces; alcohol, 2 ounces. Sig.—Given with syringe twice daily in one-half ounce doses.

This treatment I have often found to actually relax the muscles for a time, then again, would find them in the former rigid condition.

After the fourteenth or sixteenth day I generally consider the patient out of danger, but I still continue with this treatment six or eight days. Then daily doses of vegetable tonics, with nux

[1] Read before the annual meeting of the Pennsylvania State Veterinary Medical Association, March, 1898.

vomica and bromide of potassium, as there is in some cases a fear or nervousness for some time after.

Treated a gray, eight-year-old gelding some time ago. Thought it would recover. Found no wound or external cause; died the eighth day, and examination after death proved an internal injury.

Several months ago was called upon to visit a black gelding, eight years old, and found a wound just inflicted by the shaft of the wagon penetrating the scapular ulnarius muscles to the depth of several inches. Kept the horse in a box-stall five or six days. Wound nearly healed; swelling subsided; the animal appeared all right, was hitched and driven six or eight miles. About three days afterward I was called in, as the owner said the horse appeared stiff; found him very nervous and excited; could scarcely approach him. Found all the symptoms of tetanus, with the exception of the membrana nictitans not covering the eye as in all cases, and the tongue protruding from the mouth, not swollen, but for one inch was perfectly dark. Upon gently pushing it back into the mouth, it would remain for some time. No other dark or purple spots found. The animal died in about thirty-eight hours.

